# The association between contraceptive use and desired number of children among sexually active men in Zambia

**DOI:** 10.1186/s12889-023-16750-0

**Published:** 2023-09-20

**Authors:** Bwalya Bupe Bwalya, Mwewa E. Kasonde, James Nilesh Mulenga, Chabila Christopher Mapoma, Nayunda Wamunyima, Billy Siamianze, Obinna Onukogu

**Affiliations:** 1 Mulungushi University, Kabwe, Zambia; 2 Department of Economics, School of Social Science, Mulungushi University, Kabwe, Zambia; 3 Department of Population Studies, School of Humanities and Social Sciences, The University of Zambia, Lusaka, Zambia; 4 Department of Political and Administrative Studies, School of Humanities and Social Sciences, The University of Zambia, Lusaka, Zambia; 5 Department of Social Development Studies, School of Social Science, Mulungushi University, Kabwe, Zambia

**Keywords:** Males, Contraceptive use, Desired number of children, Zambia

## Abstract

**Background:**

Contraceptive methods have been used to space births, but also to limit a couple’s desired number of children. Efforts of family planning programmes have mainly concentrated on females, even though males tend to have large say on the desired number of children a couple should have. In our study, we sought to determine linkages between contraceptive use and desired number of children, as well as associated demographic and socio-economic characteristics, among sexually active males in Zambia.

**Methods:**

The main outcome variable of interest was desired number of children as measured by ideal number of children which is a count variable. Data for this paper was the male dataset from the 2018 Zambia Demographic and Health Survey, a cross-sectional national survey. Binary logistic regression was performed to determine odds ratios of contraceptive use by selected characteristics of sexually active males. Multivariate Poisson Regression Model was used to establish factors associated with desired number of children.

**Results:**

Age of men (20–29, 30–39 and 40–49 years), residence in rural areas, wealth quintile, Protestant or Muslim religious affiliation, media exposure, and having discussed family planning with a health worker in the last few months prior to the survey were associated with contraceptive use. Sexually active males who reported using any contraception method reported 3% less desired number of children compared to those who were not using any method. Older males (age group 30–49 years), resident in rural areas, with primary education, married, employed, Protestant religion, and those labelling women who use contraceptives “as promiscuous” had more desired number of children.

**Conclusions:**

There were minimal differences in the desired number of children among males who reported using and not using any contraceptive method. Strategies aimed at encouraging contraception use should cover all categories of males to achieve universal involvement of men in family planning in Zambia. Future research may consider combining both qualitative and quantitative methods to look holistically at the demographic, socio-economic and cultural factors associated with non-contraception use and desired number of children among sexually active men in Zambia.

## Introduction

Sub-Saharan Africa (SSA) remains one of the regions in the world with high fertility compared to other regions. On average, the United Nations Department for Economic and Social Affairs (UNDESA) found that a woman in SSA was found to have 4.6 children in her reproductive life, a figure twice as high as the global average of 2.4 children [1]. Modest declines in birth rates have slowed even further over the last few decades, causing concerns as to whether women and men have access to contraceptives in Africa and SSA in particular [2]. Lack of, or inconsistent and incorrect, contraceptive use is associated with unintended or mistimed pregnancies [3], unsafe abortions [3, 4] and maternal mortality [3].

To overcome this, most countries have been implementing family planning (FP) and sexual reproductive health (SRH) related services, including contraceptives [5]. These services allow women and men to acquire the knowledge that helps them to decide on the number of desired children they would want to have and their spacing. Like other countries within SSA such as Zimbabwe and Rwanda, Zambia has implemented some of the best FP and SRH programmes—with minor challenges [5]. Therefore, FP remains a fundamental area of action in Zambia if it is to lower its fertility levels [6]. The 2018 Zambia Demographic and Health Survey (ZDHS) shows that knowledge of any method of contraception among married men aged 15–59 years is almost universal (99.9%). Though it did not report the percentage of sexually active men using contraceptives, findings indicate that in Zambia, men want to have slightly more children than women, 4.9 children and 4.6 children, respectively [7]. Moreover, studies conducted elsewhere indicate that most men use contraception for spacing rather than from limiting the desired number of children [8–11].

Evidence on contraceptive use and desired number of children among sexually active men in Zambia is not yet clear, as studies have either concentrated on one of the two outcomes of this study and usually among females [12–15]. Further, studies to understand the role that men play in contraceptive use and the desired number of children have received limited attention [16–18].

Further, there is a perceived negative attitude, mostly by men, towards SRH behaviour presumably resulting from unmet need for and use of contraceptives [16, 18]. In addition, there exists strong cultural beliefs that matters of contraception are a concern for women and not men [19, 20]. This therefore tends to deter sexually active men from seeking access to contraceptives and health information, thereby exposing not only themselves but also their partners to unintended pregnancy [21, 22].

This scenario, if left unchecked, may exacerbate the existing challenges of high fertility, maternal and infant mortality as well as high-unwanted pregnancies and abortions. Whereas SRH services are available in most health facilities in Zambia, they are perceived to be for women [23, 24]. Contraceptive options for men are limited to male condoms and vasectomies, with the latter being shunned due to its invasive nature. This makes it almost impossible for men to access contraceptive options to “merely” limit the number of desired children [25, 26].

Before this study, no literature was found on research conducted in Zambia on sexually active men contraceptive use and the link it has to desired number of children. Most of the studies conducted around this topic have concentrated on women’s reproductive health and FP services utilization [18, 27]. Moreover, many times, studies on male contraceptive use and desired number of children have been conducted in middle income countries [26, 28]. Such studies have seldom been conducted in Zambia, a country where sexually active men still have insurmountable influence on the desired number of children a couple must have. To build on this existing knowledge this study sought to examine the association between contraceptive use and the desired number of children among sexually active men aged 15–59 years in Zambia.

## Methodology

### Data source

This study utilized the male recode dataset (ZMMR71FL) from the 2018 Zambia Demographic and Health Survey (ZDHS), the sixth survey conducted in Zambia since 1992. DHSs are surveys with a nationally representative sample designed to produce estimates on a range of basic demographic and health indicators at national and subnational levels, with further disaggregation to have estimates in rural and urban settings.

### Sampling design and procedure

The 2018 ZDHS sample was drawn from enumeration areas (EAs) lists captured during the 2010 Census of Population and Housing (CPH). The EA frame of the 2010 CPH was updated to accommodate realignments of districts and constituencies where some districts and constituencies were demarcated to form new ones. DHS use a stratified two-stage cluster sample design. Sample clusters consisting of EAs selected with a probability proportional to their size within each sampling stratum. About 545 clusters (198 in urban areas and 347 in rural areas) were selected at first stage while the second stage involved systematic sampling of households in all selected clusters (EAs). An average of 133 households were found during household listing from which 25 households were selected using an equal probability systematic selection process, to obtain a total sample size of 13,625 households.

All men aged 15–59 years who were either permanent residents of the households or visitors present in the households on the night before the survey were eligible to be interviewed. As noted already, all variables of analysis were based on the man’s recode dataset. The Man’s Questionnaire collected information from all eligible men at household level on topics such as background characteristics, reproduction, contraception, marriage and sexual activity, fertility preferences, employment and HIV/AIDS. Prior to conducting an interview during the 2018 ZDHS, informed consent was obtained from all the selected eligible men for interviews. For this current study, the authors obtained approval from the DHS Program to use the 2018 ZDHS male dataset.

### Target population and sample size

For this study a subsample, which included all sexually active men was drawn. During the 2018 ZDHS, 12,132 males aged 15–59 years were interviewed on various topics. However, our study excluded the following men: never had sex (1,803); no sex in the 12 months prior to the survey (657); male and female sterilization methods of contraception (55); male or partner declared infecund (111) and non-numeric response on ideal number of children (183). Therefore, a total of 9,378 unweighted sample and a weighted sample of 9,333 men was included as the sample size for this study.

### Study variables

#### Dependent variables

The study had one main outcome variables: Desired number of children as measured by ideal number of children. However, before doing this, the study further wanted to establish the prevalence of contraceptive use among sexually active males and the factors associated with its use. To do this, contraceptive use was generated as a dichotomous variable based on current contraceptive use where the response of “0” meant “no use of any method” and “1” meant “using any method of contraception”. All missing cases and those who reported “did not know” response was excluded from the analysis. The main outcome variable *desired number of children* as measured by “ideal number of children” was collected as a count variable; as in prefer 1, 2, 3, 4, or so children. In the regression model, desired number of children was analysed as a count and was not recoded.

### Independent variables

The explanatory variables used in this study included: Age of men coded as 15–19 (reference category), 20–29, 30–39, 40–49, 50–59. Place of residence categorized as urban (reference category) and rural. Education level categorized into no education, primary, secondary, higher education (reference category). Wealth index was used as a proxy measure of household’s wealth recoded as poorest, poorer, middle, richer and richest wealth quintiles (reference category). Current marital status was in three categories: never married (reference category), currently married and formerly married. Other explanatory variables included, religion, discussing family planning with a health worker, media exposure, and attitudes towards contraception use by women amongst males.

### Statistical analysis

Analysis of data was done using Stata version 15. For both explanatory and predictor variables, descriptive statistics were performed. In order to consider the complex nature of the sampling process and ensuring standardization of estimates, the data analysis procedure included weighting the data using the Stata command “svy”. To determine associations between independent and dependent variables, Chi-square was used. Before performing multiple regressions, multicollinearity tests were done among independent variables to detect presence of collinearity. Considering that the outcome variable, *desired number of children* as measured by “ideal number of children” was concentrated over few small discrete values, we used count data regressions. Specifically, we employed the Poisson Regression model to establish factors associated with desired number of children among men 15 – 59 years. The Adjusted Incidence Rate Ratio (AIRR) results with *p*-values and 95% confidence intervals (CI) were considered.

## Results

### Distribution of respondents by characteristics

In Table 1, results show that about five in every ten males (50%) were using contraceptive methods (modern or traditional). The age group 20–29 years had the highest percentage of the study population (34%) while the lowest was in the age group 50–59 years (8%). Fifty-nine percent of males resided in rural areas, 48% had attained secondary education; 21% and 22% respectively were in the richest and richer wealth quintiles. Sixty-six percent of the men were currently married. About nine in ten (85%) males were currently working, and 79% were of the Protestant religion. Results show that about 17% of men reported to have discussed family planning with a health worker. Thirty-eight percent of the men had exposure to media. Table 1 also shows that only 24% of men agreed to the statement “contraception is a woman's business, a man should not worry”, while about 39% of the men agreed to the statement “women who use contraception become promiscuous.”

**Table 1:** Percentage distribution of men by demographic, socio-economic and other characteristics

Variable	Percent	Count
**Contraceptive use**		
None	50.2	4,687
Any contraception	49.8	4,646
**Age group**		
15 – 19	11.1	1,038
20 – 29	34.0	3,170
30 – 39	26.8	2,502
40 – 49	19.7	1,840
50 – 59	8.4	783
**Type of place of residence**		
Urban	41.4	3,862
Rural	58.6	5,471
**Educational level**		
No education	4.3	405
Primary	38.6	3,598
Secondary	47.8	4,461
Higher	9.3	870
**Wealth index**		
Poorest	17.9	1,675
Poorer	18.6	1,740
Middle	20.4	1,906
Richer	21.6	2,020
Richest	21.3	1,992
**Marital status**		
Never married	29.8	2,783
Currently married	65.9	6,150
Formerly married	4.3	400
**Employment status**		
Not working	15.2	1,416
Currently working	84.8	7,917
**Religion**		
Catholic	19.5	1,824
Protestant	78.9	7,361
Muslim	0.6	53
Other	1.0	95
**Discussed FP with health worker in last few months**		
No	83.0	7,745
Yes	17.0	1,588
**Media exposure**		
No exposure	61.6	5,753
Exposed	38.4	3,580
**Contraception is woman's business, man should not worry**		
Disagree	73.3	6,846
Agree	24.1	2,246
Don't know	2.6	242
**Women who use contraception become promiscuous**		
Disagree	58.4	5,450
Agree	38.6	3,602
Don't know	3.0	282
Total	100.0	9,333

### Contraceptive use and sexually active men’s characteristics

Table 2 shows results of the chi-square test between contraceptive use and men’s demographic, socioeconomic, and other characteristics. Results show that contraceptive use is significantly related (*p* < 0.05) with the following men’s characteristics: age group, type of place of residence, highest education level attained, wealth status, marital status, religion, discussion of family planning with health worker, access to media and the statement “contraception is a woman's business, a man should not worry”. However, employment status (working or not working) and the perception that “women who use contraception become promiscuous” were not significantly related with contraceptive use among men (*p* > 0.05).

**Table 2:** Contraceptive use among sexually active men by demographic, socio-economic and other characteristics

Variable	Contraceptive Use					
	**None**		**Yes, used any method**		**Number**	***P*-value**
	**%**	**CI**	**%**	**CI**		
**Age group**						0.000
15 – 19	55.8	[51.5,60.0]	44.2	[40.0,48.5]	1,038	
20 – 29	47.2	[44.8,49.6]	52.8	[50.4,55.2]	3,170	
30 – 39	45.8	[43.3,48.3]	54.2	[51.7,56.7]	2,502	
40 – 49	51.6	[48.6,54.6]	48.4	[45.4,51.4]	1,840	
50 – 59	66.0	[62.1,69.8]	34.0	[30.2,37.9]	783	
**Type of place of residence**						0.008
Urban	47.4	[44.4,50.5]	52.6	[49.5,55.6]	3,862	
Rural	52.2	[50.5,53.9]	47.8	[46.1,49.5]	5,471	
**Educational level**						0.000
No education	57.1	[50.9,63.2]	42.9	[36.8,49.1]	405	
Primary	57.8	[55.5,60.2]	42.2	[39.8,44.5]	3,598	
Secondary	45.9	[43.7,48.1]	54.1	[51.9,56.3]	4,461	
Higher	37.7	[32.3,43.5]	62.3	[56.5,67.7]	870	
**Wealth index**						0.000
Poorest	59.8	[57.1,62.5]	40.2	[37.5,42.9]	1,675	
Poorer	53.6	[50.9,56.3]	46.4	[43.7,49.1]	1,740	
Middle	49.5	[47.1,51.9]	50.5	[48.1,52.9]	1,906	
Richer	49.0	[45.3,52.7]	51.0	[47.3,54.7]	2,020	
Richest	41.1	[38.0,44.3]	58.9	[55.7,62.0]	1,992	
**Marital status**						0.007
Never married	46.9	[44.4,49.5]	53.1	[50.5,55.6]	2,783	
Currently married	51.8	[49.9,53.7]	48.2	[46.3,50.1]	6,150	
Formerly married	48.8	[42.2,55.4]	51.2	[44.6,57.8]	400	
**Employment status**						0.370
Not working	48.5	[44.9,52.1]	51.5	[47.9,55.1]	1,416	
Currently working	50.5	[48.6,52.4]	49.5	[47.6,51.4]	7,917	
**Religion**						0.003
Catholic	53.9	[50.5,57.2]	46.1	[42.8,49.5]	1,824	
Protestant	49.3	[47.7,50.9]	50.7	[49.1,52.3]	7,361	
Muslim	37.1	[25.8,50.0]	62.9	[50.0,74.2]	53	
Other	59.6	[47.6,70.6]	40.4	[29.4,52.4]	95	
**Discussed FP with health worker in last few months**						0.000
No	51.8	[50.1,53.4]	48.2	[46.6,49.9]	7,745	
Yes	42.7	[39.7,45.8]	57.3	[54.2,60.3]	1,588	
**Media exposure**						0.000
No exposure	53.8	[52.1,55.5]	46.2	[44.5,47.9]	5,753	
Exposed	44.5	[41.8,47.3]	55.5	[52.7,58.2]	3,580	
**Contraception is woman's business; man should not worry**						0.000
Disagree	48.8	[47.2,50.4]	51.2	[49.6,52.8]	6,846	
Agree	53.7	[50.8,56.5]	46.3	[43.5,49.2]	2,246	
Don't know	58.1	[51.6,64.4]	41.9	[35.6,48.4]	242	
**Women who use contraception become promiscuous**						0.449
Disagree	50.0	[48.1,52.0]	50.0	[48.0,51.9]	5,450	
Agree	50.2	[48.0,52.3]	49.8	[47.7,52.0]	3,602	
Don't know	54.5	[47.9,60.8]	45.5	[39.2,52.1]	282	
**Total**	50.2	[48.7,51.8]	49.8	[48.2,51.3]	9,333	

### Factors associated with contraceptive use among sexually active men in Zambia

Table 3 shows Adjusted Odds Ratios (AORs) between various explanatory variables and contraceptive use among men. The odds of men using contraception were 49%, 75% and 43% higher among men aged 20 – 29, 30 – 39 and 40 – 49 years compared with those aged 15 – 19 years, respectively. Men whose place of residence was rural areas had 38% higher odds of using contraception compared with those in urban areas.

**Table 3:** Adjusted odds rations on the factors associated with contraceptive use among sexually active men in Zambia

Variable	Contraceptive Use	
	**AORs**	**CI**
**Age group**		
15 – 19	1	
20 – 29	1.49***	1.18—1.89
30 – 39	1.75***	1.32—2.32
40 – 49	1.43*	1.08—1.88
50 – 59	0.77	0.56—1.05
**Type of place of residence**		
Urban	1	
Rural	1.38***	1.18—1.62
**Educational level**		
No education	1	
Primary	0.98	0.75—1.28
Secondary	1.31	0.97—1.77
Higher	1.50	0.99—2.29
**Wealth index**		
Poorest	1	
Poorer	1.26**	1.09—1.45
Middle	1.49***	1.28—1.73
Richer	1.62***	1.33—1.98
Richest	2.02***	1.60—2.55
**Marital status**		
Never married	1	
Currently married	0.76**	0.63—0.91
Formerly married	0.87	0.61—1.24
**Employment status**		
Not working	1	
Currently working	0.87	0.73—1.04
**Religion**		
Catholic	1	
Protestant	1.23**	1.07—1.41
Muslim	2.44**	1.38—4.33
Other	0.98	0.58—1.65
**Discussed FP with health worker in last few months**		
No	1	
Yes	1.41***	1.22—1.64
**Media exposure**		
No exposure	1	
Exposed	1.21**	1.07—1.38
**Contraception is woman's business, man should not worry**		
Disagree	1	
Agree	0.88*	0.77—1.00
Don't know	0.81	0.57—1.16
**Women who use contraception become promiscuous**		
Disagree		
Agree	1.09	0.97—1.22
Don't know	1.11	0.79—1.55

^***^ *p* < 0.001, ** *p* < 0.01, * *p* < 0.05

Contraception use increased with better wealth index. It however was lower among currently married men (24%) than among those that had never been married. The odds of contraception use were higher among Protestants and Muslims (23% and 44%) than among Catholics. Results further show that men who discussed family planning issues with a health worker had higher odds (41%) of contraception use than those who did not. Besides, Table 3 also shows that, contraception use among men was higher among those exposed to media (21%) compared with those who were not.

### The association between contraceptive use and desired number of children among sexually active men in Zambia

Table 4 presents unadjusted incident rate ratio (UIRR) for the association between the desired number of children and contraceptive use among men in Zambia. The UIRR for men who used any contraception was 0.91 times the incident rate for those who reported not using any contraception.

**Table 4:** Unadjusted Incident rate ratio of the association between contraceptive use and desired number of children among sexually active men in Zambia

Variable	IRR	CI
**Contraceptive use**		
None	1	
Used any contraception	0.91***	0.88—0.94
Constant	5.52***	5.37—5.68

^***^ *p* < 0.001, ** *p* < 0.01, * *p* < 0.05

### Factors associated with desired number of children among sexually active men in Zambia

Results in Table 5 show that the adjusted incident rate ratio (AIRR) for men who used any contraception was 0.96 times the incident rate for those who reported not using any contraception. Men in the age group 20–29, 30–39, 40–49 and 50–59 had 0.93, 1.05, 1.20- and 1.31-times incident rates of those in the age group 15–19, respectively. The incident rate for men who resided in rural areas was 1.12 times that for men who resided in urban areas. Men with primary and higher education had incident rates of 1.07 and 0.87 that for men with no education, respectively. Regarding wealth status, men in middle, richer and richest households had 0.93, 0.91 and 0.85-times incidence rates compared with the poorest households, respectively.

**Table 5:** Adjusted incident rate ratio for the factors associated with desired number of children among sexually active men in Zambia

Variable	AIRR	CI
**Contraceptive use**		
None	1	
Used any contraception	0.96***	0.94—0.98
**Age group**		
15 – 19	1	
20 – 29	0.93**	0.89—0.97
30 – 39	1.05*	1.00—1.11
40 – 49	1.20***	1.14—1.26
50 – 59	1.31***	1.23—1.39
**Type of place of residence**		
Urban	1	
Rural	1.12***	1.05—1.18
**Educational level**		
No education	1	
Primary	1.07**	1.02—1.13
Secondary	0.98	0.93—1.04
Higher	0.87***	0.80—0.93
**Wealth index**		
Poorest	1	
Poorer	1.00	0.97—1.04
Middle	0.93***	0.89—0.97
Richer	0.91*	0.83—0.99
Richest	0.85***	0.78—0.92
**Marital status**		
Never married	1	
Currently married	1.09***	1.06—1.13
Formerly married	1.00	0.94—1.07
**Employment status**		
Not working	1	
Currently working	1.05**	1.02—1.09
**Religion**		
Catholic	1	
Protestant	1.04*	1.01—1.07
Muslim	1.00	0.87—1.14
Other	1.07	0.89—1.28
**Discussed family planning with health worker in last few months**		
No	1	
Yes	1.02	0.99—1.05
**Media exposure**		
No exposure	1	
Exposure	0.98	0.95—1.00
**Contraception is woman's business; man should not worry**		
Disagree	1	
Agree	0.96*	0.93—1.00
Don't know	0.92*	0.85—0.98
**Women who use contraception become promiscuous**		
Disagree	1	
Agree	1.05**	1.01—1.08
Don't know	1.01	0.94—1.09

^***^p < 0.001, ^**^p < 0.01, ^*^p < 0.05

Currently married men had 1.09 times incident rates than those who have never been married. Results in Table 5 also show that the incident rate for men who were in employment was 1.05 times those who were not in employment. The incident rate for men whose religion was Protestant was 1.04 times the incident rate for Catholics. Men who agreed and those who said they do not know whether “contraception is a woman’s business” had 0.96- and 0.92-times incident rate those who disagreed, respectively. Similarly, men who think “women who use contraceptives as promiscuous” had 1.05 times the incident rates than those who disagreed.

## Discussion

It is widely recognized that consistent and correct use of contraceptives by couples can help space and or limit the number of children. This paper aimed at examining the association between contraceptive use and the desired number of children among sexually active males aged 15–59 years in Zambia. Findings from this present study showed that use of any contraceptive method among sexually active males is low in Zambia (50%). This result is different to prior research conducted in Myanmar, where it was established that only about 39% of the males were using modern contraception [29]. This might be because, though currently the FP services have also started targeting males, especially among couples, little is being done to increase awareness-raising campaigns that traditionally have targeted men [30]. These campaigns will continue to be critical owing to the very important traditional family roles men are said or expected to play within the family.

While the forgoing is true, others have argued that the desire for boy-children and accusing female partners of salacious behaviour contributes to men not wanting to use contraceptives [16, 31]. Others have also argued that in Africa most people would love to have lower numbers of children with acceptable spacing. One of the challenges faced, however, is that FP services are limited in supply and usually unavailable when needed [32].

It is worth noting from this current study that, there exists a significant relationship between males using contraceptives and desired number of children as revealed by both the unadjusted and adjusted IRRs. Nonetheless, there was minimal differences in the desired number of children between men who reported using any contraception method and those not using. Studies conducted elsewhere reveal that patrilineal traditions tend to perpetuate value for children and encourages large family size [30]. Therefore, what influences decisions is not mostly a factor of use or non-use of contraception but what the larger family feels and expects. Such undertones seem to be applicable to the case in Zambia, given these results.

Younger males reported having a lower desired number of children compared to those in the age group 45 – 49. This is not an “out of range” result as it were. It seems, younger males when compared to the older ones, may not have a concrete disclosure of what they would perceive as a desired number of children. This is even though age is a key factor that influences fertility. Some more recent studies confirm that a man’s age affects conception in women, however, male potency to impregnate a woman dwindles significantly after age 40 due to several reasons but among them is low sperm quality [33].

Compared to other studies of a similar nature, this study reveals that the desired number of children for males residing in rural areas was 8% higher compared to those who reside in urban areas. Various reasons can be advanced for this observed phenomenon. Firstly, men in rural areas are culturally sensitive and therefore are more likely to prefer more children than their urban counterparts [34]. In many communities of Africa, Middle East and parts of Latin America, children have economic value since they are a cheap source of labour, wealth, and financial security [30] Secondly, contraceptive choices in rural areas are significantly limited for both men and women due to low supply and in some cases, long distances to access them [35]. With such challenges, it becomes a costly affair and a luxury for males to find contraceptives, let alone use contraceptives [30].

Our study shows that men who had attained secondary and tertiary education have lower relative risk of desired number of children compared with those with no education. This is like studies [29, 36] that found that men with a higher level of education were less likely to desire more than two children compared with men with no education. Moreover, low levels of education among males are likely to lead to enhanced misconceptions about contraceptive use and assumed side effects. Besides, it can equally lead to limitations in accessing information that can change their cultural construct whose result is a large family size. Similarly, men from households classified as poor and middle in terms of wealth status were more likely to have higher desired number of children compared with men from households considered rich. This finding is not surprising as most households in Zambia with more children are those in the poor wealth group and in rural areas [37].

In the African context, men who are employed are seen as being wealthy and usually tend to have more partners and children because, generally, they can support their children and families [38]. Findings from our study reveal that men who are currently employed had higher chances of increasing their desired number of children compared to the unemployed. This can be explained by the fact that, in a country where unemployment is low for women than that of men, women would rather be associated with men who are both currently working and have a stable income. Furthermore, these men tend to be the main decision maker regarding the desired number of children, as they will be able to control their women due to their employment status [39].

Religion plays a major role in determining the number of desired children a couple may wish to have. Our study reveals that Protestants had a 4% higher chance of having more children than Catholics. This result is at variance with what [34] had been found in a study in Kenya among Muslims where they had higher odds of having more children than Catholics. Moreover, it is documented that religion amplifies the idea of more children [30].

Media exposure is critical for informing, educating, and communicating accurate and relevant information on fertility and related topics. Therefore, access and utilisation of such information may be significant in reducing unwanted fertility observed in Zambia. Findings from our study, show that there are no significant differences in preference for children between males who had no access to media and those who had access. This deviates from other studies where the media is suggested to influence decision making with regards to fertility and that men who are rarely engaged as principal targets for Information, Education and Communication (IEC) sensitizations on contraceptives and fertility related issues often prefer more children [34]. Moreover, it is argued that when men have more information, there is a high chance that their awareness is enhanced, and they become active participants of fertility regulation thereby contributing to reduction in unintended fertility as there is a convergence of the means and social cultural changes [40].

Attitudes towards contraception use does affect its use or non-use. Studies relative to ours found similar results with women who tend to utilize family planning services mostly accused of being adulterous and, in turn, avoid becoming pregnant for their male partners [34].

### Study limitations

The DHS data can only estimate associations and not causation as such, what is presented herein are associations which may not respond to how or why certain results appear in the manner they do. Furthermore, estimates of any contraceptive use may be underestimated or overestimated because of recall bias given the retrospective nature of the data used. Men may not always be aware that their partners are taking a method, such as pills, injectable, implants, or an intrauterine device, when no prior discussions have been had or when a method is not something a man would approve of.

## Conclusion

Core to reducing the desired number of children and ultimately fertility in a country with high fertility is the critical role that men play in the use of contraceptives if the desired number of children is to be reduced. Contraception use is highly influenced by demographic and socio-economic characteristics of males. Importantly, this study has revealed that any contraception usage alone is not a major determinant in men’s desired number of children. Both demographic and socio-economic factors have a major influence to provide guidance on increased male involvement in family planning activities among sexually active men in Zambia.

There still exist some ideas among the Zambian populace that men assumed to be rich should have a higher desired number of children because they can take care of them. This is coupled with misconceptions that women who use contraception are promiscuous. Therefore, to enhance uptake of contraceptive use and reduce these misconceptions, there is need for enhanced FP and IEC for this group of males. Besides, strategies aimed at encouraging contraception use should cover all categories of men to achieve universal involvement in FP in Zambia. Future research may consider combining both qualitative and quantitative aspects to have a holistic picture of how demographic, socio-economic, and cultural factors would affect non-contraception use and desired number of children among sexually active men in Zambia.

## Authors’ information

B.B.B works as a lecturer of Demography and Research Coordinator-Directorate of Research and Postgraduate Studies at Mulungushi University in Zambia. B.B. B’s research interests include child health, nutrition, and mortality; maternal health and nutrition; HIV and AIDS; adolescent reproductive health, and fertility.

M.E.K is a lecturer of Demography at the University of Zambia. M.E. K’s research interests include maternal health and nutrition; family planning; fertility; mortality; child health and nutrition; and monitoring and evaluation.

J.N.M is currently a lecturer and researcher of Economics at Mulungushi University in Zambia. His main research interests are on health care utilization and outcomes, trade policy and development, and unemployment.

N.W is a lecturer of Public Administration at the University of Zambia. N. W’s research interests include human rights (including reproductive rights), political culture, gender equality, citizens’ participation, and governance.

O.O. is a governance expert, teaching Development Studies at Mulungushi University in Zambia. O. O’s research interests include agrarian studies, informality, pro-poor policies, poverty and inequality, social structure, fertility, behavioural change, civil society, and governance.

B.S is a vibrant career Military Officer based with the Zambia Air Force and has high academic aspirations. B. S’s core research interests include health care management and administration.

C.C.M (PhD) is a Senior Lecturer in the Department of Population Studies at the University of Zambia. C.C. M’s research interests include population aging; civil registration; maternal health and nutrition; fertility; mortality; and child health and nutrition; and monitoring and evaluation.

## Data Availability

The dataset can be accessed following clearance after written request at https://dhsprogram.com/Data/. Dataset and materials used in this paper are available on request from the corresponding author (bwalya83@gmail.com).

## Abbreviations

AIDS: Acquired Immunodeficiency Syndrome
AORs: Adjusted Odds Ratios
CI: Confidence Interval
CPH: Census of Population and Housing
DHS: Demographic and Health Survey
EA: Enumeration Area
EAs: Enumeration Areas
FP: Family Planning
FPC: Family Planning and Contraceptive
HIV: Human Immunodeficiency Virus
ICPD: International Conference on Population and Development
IEC: Information Education and Communication
IVs: Independent Variables
MoH: Ministry of Health
SDGs: Sustainable Development Goals
SRH: Sexual and Reproductive Health
TFR: Total Fertility Rate
UIRR: Unadjusted Incident Rate Ratio
UNDESA: United Nations Department of Economic and Social Affairs
UN: United Nations
ZDHS: Zambia Demographic and Health Survey
ZamStats: Zambia Statistics Agency
